# Influence of size, volume concentration and aggregation state on magnetic nanoparticle hyperthermia properties *versus* excitation conditions†

**DOI:** 10.1039/d3na00709j

**Published:** 2024-02-14

**Authors:** Riccardo Ferrero, Marta Vicentini, Alessandra Manzin

**Affiliations:** a Istituto Nazionale di Ricerca Metrologica (INRiM) Strada delle Cacce 91 10135 Torino Italy r.ferrero@inrim.it a.manzin@inrim.it

## Abstract

Treatment planning in magnetic hyperthermia requires a thorough knowledge of specific loss power of magnetic nanoparticles as a function of size and excitation conditions. Moreover, in biological tissues the magnetic nanoparticles can aggregate into clusters, making the evaluation of their heating performance more challenging because of the magnetostatic dipole–dipole interactions. In this paper, we present a comprehensive modelling analysis of 10–40 nm sized spherical magnetite (Fe_3_O_4_) nanoparticles, investigating how their heating properties are influenced by magnetic field parameters (peak amplitude and frequency), and by volume concentration and aggregation state. The analysis is performed by means of an in-house micromagnetic numerical model, which solves the Landau–Lifshitz–Gilbert equation under the assumption of single-domain nanoparticles, including thermal effects *via* a Langevin approach. The obtained results provide insight into how to tune hyperthermia properties by varying magnetic nanoparticle size, under different excitation magnetic fields fulfilling the Hergt–Dutz limit (frequency between 50 kHz and 1 MHz, and peak amplitude between 1 kA m^−1^ and 50 kA m^−1^). Special attention is finally paid to the role of volume concentration and aggregation order, putting in evidence the need for models able to account for stochasticity and clustering in spatial distribution, to accurately simulate the contribution of magnetostatic dipole–dipole interactions in real applications.

## Abstract 2

A micromagnetic model is used to evaluate the heating properties of Fe_3_O_4_ nanoparticles *versus* size, concentration, aggregation order, and AC magnetic field parameters. The obtained data can guide nanoparticle design in view of hyperthermia application.

## Graphical abstract

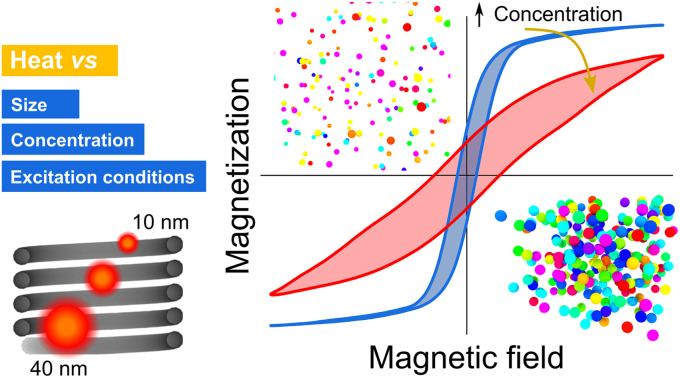

## 1 Introduction

Magnetic nanoparticles (NPs) can find application in different fields of biomedicine,^1–5^ where they can be employed in therapeutics, as mediators for heat-assisted drug release and magnetic hyperthermia, and in diagnostics, as contrast agents in magnetic resonance imaging (MRI) and tracers in magnetic particle imaging (MPI). In this panorama, magnetic hyperthermia has been largely investigated as an adjuvant for improving the efficacy of radiotherapy and chemotherapy in cancer treatment.^6–9^ One of its potential advantages over standard hyperthermia techniques is the possibility of obtaining a more localized and controlled heat release in the target region. This can be achieved by exciting the magnetic NPs with an alternating current (AC) magnetic field, with frequencies in the range between 50 kHz and 1.2 MHz.^10^ The field induces a cyclic response of the NP magnetization configuration, which results in power dissipation due to hysteresis losses.^11^

However, AC magnetic fields can also cause undesired eddy current effects, with the possible occurrence of non-selective heating of healthy tissues, as observed in preclinical tests on mice and rats,^12^ and corroborated by *in silico* models.^13^ Since the specific heating power produced by eddy currents is proportional to the square of the current path radius and to the square of the product of the peak amplitude *Ĥ*_a_ and frequency *f* of the AC magnetic field, limits on *Ĥ*_a_ × *f* are conventionally imposed to reduce the risks for safety, as a function of the size of the body part exposed to the field. A first upper limit was established for adult human thorax by Atkinson and Brezovich in 1984,^14^ that is *Ĥ*_a_ × *f* ≤ 4.85 × 10^8^ A m^−1^ s^−1^. A less severe restriction, *Ĥ*_a_ × *f* ≤ 5 × 10^9^ A m^−1^ s^−1^, was suggested later by Hergt and Dutz,^15^ to be applied when treating small human body parts or small animals (*e.g.* mice), bearing in mind that exceeding this limit could be critical even for rats.^13^ Then, besides the optimization of heating properties, these constraints should be considered when designing novel magnetic NPs for magnetic hyperthermia applications. Conversely, in many studies, magnetic NPs were characterized under experimental conditions that exceed such limits, without addressing the possibility of optimizing heating properties at field amplitudes and frequencies more compatible with application on living beings. Attempts in this direction were made by proposing the use of hard magnetic NPs able to release heat under conditions even below the Atkinson–Brezovich limit.^16^

One of the indexes typically used to express the capability of magnetic NPs to release heat is the specific loss power (SLP), *i.e.* the power dissipated per unit mass of the magnetic material, which can be derived from thermometric or calorimetric measurements.^17^ The produced heat is directly correlated to the area of the magnetic NP hysteresis loop, which depends on excitation conditions^18,19^ (peak amplitude and frequency of the AC magnetic field) and NP properties (size,^20–25^ shape,^20,25–28^ material composition,^28–31^ surface coating,^32,33^ and aggregation state^34–36^). In view of the design of magnetic NPs with high SLP values, many efforts have been concentrated on the increase in the effective magnetic anisotropy constant, using materials with high uniaxial magnetocrystalline anisotropy, synthesizing NPs with strong shape anisotropy (*e.g.* rod-shaped) or exploiting NP arrangement in chains.^20,37^

In this vast panorama, besides experimental investigations, many analytical and numerical modeling approaches have been proposed to evaluate the SLP of magnetic NPs, as a function of excitation conditions and NP properties. Analytical models can provide a rapid estimation of SLP,^38–40^ but have limitations on their applicability, *i.e.* they are generally restricted to magnetic NPs with uniaxial anisotropy and uniform material properties, and to very diluted concentrations, neglecting magnetostatic dipole–dipole interactions. Recently, a more exhaustive analytical model has been proposed to include the effects of such interactions on the hysteresis losses, but the simplified assumption of magnetic NPs distributed on regular grids is considered.^41^

Monte Carlo (MC) based models^42–45^ have also been used to calculate the hysteresis loops of ensembles of non-interacting magnetic NPs or clusters of strongly interacting magnetic NPs, with the possible inclusion of exchange coupling within each of them. Despite its reliability, this approach is not free of drawbacks, since MC simulations do not generally involve the evaluation of magnetization dynamics, making more difficult the reproduction of rate-dependent hysteresis behavior that leads to the influence of *f* on the hysteresis losses. This issue can be overcome by means of kinetic MC models, which enable the simulation of both time and temperature dependence in magnetic NP hysteresis loop calculation.^46,47^ Alternatively, phenomenological magnetodynamics approaches have been successfully applied to model dynamic magnetic hysteresis and heat generation in both liquid and solid suspensions.^30^

The behavior of magnetic NPs can also be studied by numerically solving the Landau–Lifshitz–Gilbert (LLG) equation, whose spatial-time integration can allow non-uniform magnetic domain configurations and complex magnetization processes to be reproduced,^48^ like the ones appearing in disk-shaped NPs.^49^ However, full micromagnetic simulations are computationally very intensive and not suitable to describe thousands of magnetic NPs. An appropriate solution to investigate the magnetization dynamics and hysteresis losses of a large ensemble of magnetic NPs with the LLG equation is the macrospin model, valid under the assumption of single-domain magnetic NPs.^50–52^

In this work, a micromagnetic solver based on the stochastic LLG equation and on the macrospin approximation is implemented to study the response of magnetic NPs to different AC magnetic fields, under conditions fulfilling the Hergt–Dutz limit.^15^ The developed solver is able to simulate the dynamic hysteresis loop of a large number of magnetic NPs, enabling the inclusion of temperature effects, magnetostatic dipole–dipole interactions and random distribution of magnetocrystalline anisotropy axes. Here, it is applied to calculate the hysteresis losses and the SLP of magnetite (Fe_3_O_4_) NPs with a spherical shape and size between 10 and 40 nm. The analysis is performed on very diluted NPs as well as on strongly interacting NPs, with variable volume concentration and aggregation order. The results of the simulations enable us to provide indications on how increasing the SLP of spherical Fe_3_O_4_ NPs, by properly modifying the NP size and the AC magnetic field parameters.

The obtained outcomes can guide researchers working in magnetic hyperthermia in selecting the magnetic NP features and excitation conditions suitable for *in silico* and *in vivo* tests, and for possible clinical translation. Moreover, besides the evaluation of the SLP *versus* magnetic field peak amplitude and frequency, the prediction of the NP heating properties *versus* volume concentration and aggregation order can provide feedback for treatment planning and heat delivery modulation. Benefits are expected when combining magnetic hyperthermia with imaging techniques able to accurately quantify magnetic NP concentration and spatial distribution within target tissues, like magnetic particle imaging (MPI).^53^

## 2 Computational methods

The micromagnetic numerical model is developed under the assumption of single-domain magnetic NPs with a spheroidal shape, having the same material composition, and that can influence each other through magnetostatic dipole–dipole interactions. The model applied to an ensemble of magnetic NPs is based on the numerical integration of the LLG equation:1
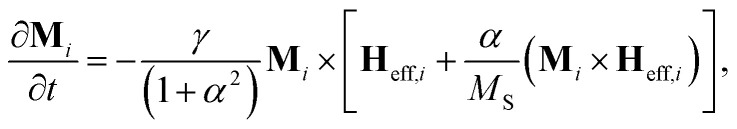
where **M**_*i*_ is the magnetization vector within the *i*-th NP, with volume *V*_*i*_ and saturation magnetization *M*_S_, *γ* is the absolute value of the gyromagnetic ratio and *α* is the damping coefficient. The effective field **H**_eff,*i*_ within the *i*-th NP is expressed as the sum of the applied external field **H**_a_, the magnetocrystalline anisotropy field **H**_an,*i*_, the internal magnetostatic field **H**_ms,*i*_, the magnetostatic interaction field **H**_dip,*i*_ resulting from dipole–dipole interactions between NPs, and the thermal field **H**_th,*i*_. The exchange field contribution is negligible, assuming that the NPs are uniformly magnetized.

In the case of cubic magnetocrystalline anisotropy (valid for Fe_3_O_4_ NPs), **H**_an,*i*_ is expressed as:2
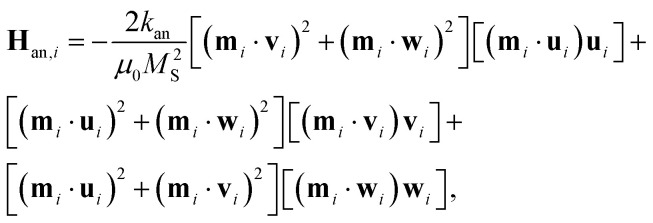
where μ_0_ is the vacuum magnetic permeability, *k*_an_ is the first-order cubic anisotropy constant and (**u**_*i*_, **v**_*i*_, **w**_*i*_) is an orthonormal triad of unit vectors parallel to the *i*-th NP anisotropy axes.

Under the assumption of spherical NPs, the internal magnetostatic field is calculated as **H**_ms,*i*_ = −**M**_*i*_/3. In the presence of shape anisotropies (*e.g.* for discoidal or elongated NPs), **H**_ms,*i*_ can be evaluated by using the analytical expressions of the demagnetizing factors for spheroidal objects.^54^

The relationship for the magnetostatic interaction field **H**_dip,*i*_ is:3
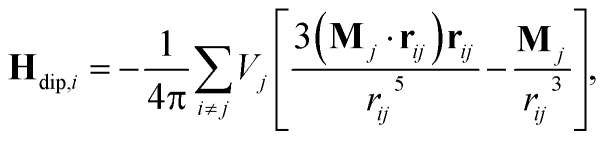
where **r**_*ij*_ is the vector from the *i*-th NP to the *j*-th one.^55^

The thermal field **H**_th,*i*_ is determined following the Langevin approach and the fluctuation-dissipation theorem, resulting in:4
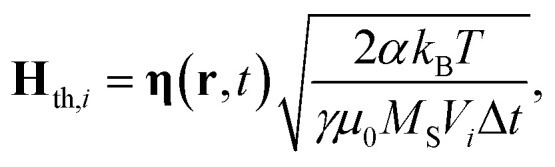
where *T* is the absolute temperature, *k*_B_ is the Boltzmann constant and Δ*t* is the time-step used in the time integration; **η**(**r**, *t*) is a stochastic vector whose components are Gaussian random numbers, uncorrelated in space and time, and with zero mean value and dispersion equal to 1.^56^

The magnetization within each NP is updated by means of a geometric time-integration scheme based on the Cayley transform, which enables us to intrinsically preserve the constraint on magnetization amplitude.^57^ The time integration is performed with a second-order scheme based on the Heun algorithm.

For each examined sample and AC magnetic field condition, we evaluate the specific energy losses, as:5
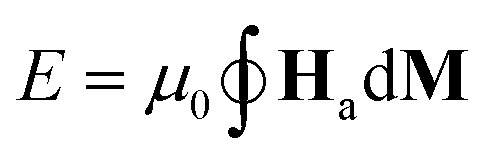


The specific loss power (SLP) is then theoretically estimated as SLP = *fE*/*ρ*, where *ρ* is the mass density of the magnetic material.

As a further improvement, the micromagnetic numerical model here developed can be extended to multicore NPs, which can be described as clusters of spherical cores approximated as magnetic dipoles, with a variable number of cores within each cluster and packing density. The simulation of these nanomaterials would require a proper implementation of the exchange interactions between the cores, as detailed in previous relevant studies.^58^

## 3 Results and discussion

In the following analysis, we investigate the influence of AC magnetic field parameters (peak amplitude *Ĥ*_a_ and frequency *f*) on the SLP of spherical Fe_3_O_4_ NPs, varying their size (between 10 and 40 nm) and their aggregation state. Parameters *Ĥ*_a_ and frequency *f* are selected to guarantee the fulfillment of the Hergt–Dutz limit,^15^ changing accordingly *Ĥ*_a_ between 1 kA m^−1^ and 50 kA m^−1^, and *f* between 50 kHz and 1 MHz.

The magnetic properties of Fe_3_O_4_ NPs are set as follows: damping coefficient *α* = 0.02; saturation magnetization *M*_s_ = 410 kA m^−1^ (being *M*_s_ typically lower for the NP size range here considered than for the bulk material);^59^ first-order cubic magnetocrystalline anisotropy constant *k*_an_ = −13.5 kJ m^−3^.^60^ The directions of the anisotropy axes are sampled from a uniform distribution on the whole solid angle. In all the simulations, the temperature is set at 300 K.

In order to validate the single-domain approximation, we perform a preliminary study with a full 3D micromagnetic approach,^61^ applied on Fe_3_O_4_ nanospheres with variable size. The results are reported in Fig. S1 of the ESI,† for a nanosphere with a diameter of 40 nm, discretized with 2.5 nm cubic cells. The dynamic hysteresis loops calculated for two directions of the applied magnetic field are typical of a reversal process dominated by a quasi-coherent rotation of the magnetization (Fig. S1a†). The irreversible jump corresponds to a transition from one “onion” state to another one of opposite polarity (Fig. S1b†). In particular, the magnetization vectors are characterized by a small canting towards the nanosphere surface, before and after the transition. Then, the Fe_3_O_4_ nanospheres can be well approximated as single dipoles, also for the largest considered size, confirming the validity of the simplified “macrospin” model for the entire range of investigated diameters.

### 3.1. Non-interacting magnetic nanoparticles: role of size and magnetic field parameters

In this sub-section, we study how magnetic NP size and AC magnetic field parameters affect the hysteresis losses, focusing on non-interacting NPs. We consider samples consisting of 2000 equal Fe_3_O_4_ NPs, randomly distributed in the space with respect to the applied magnetic field direction.

**Fig. 1:**
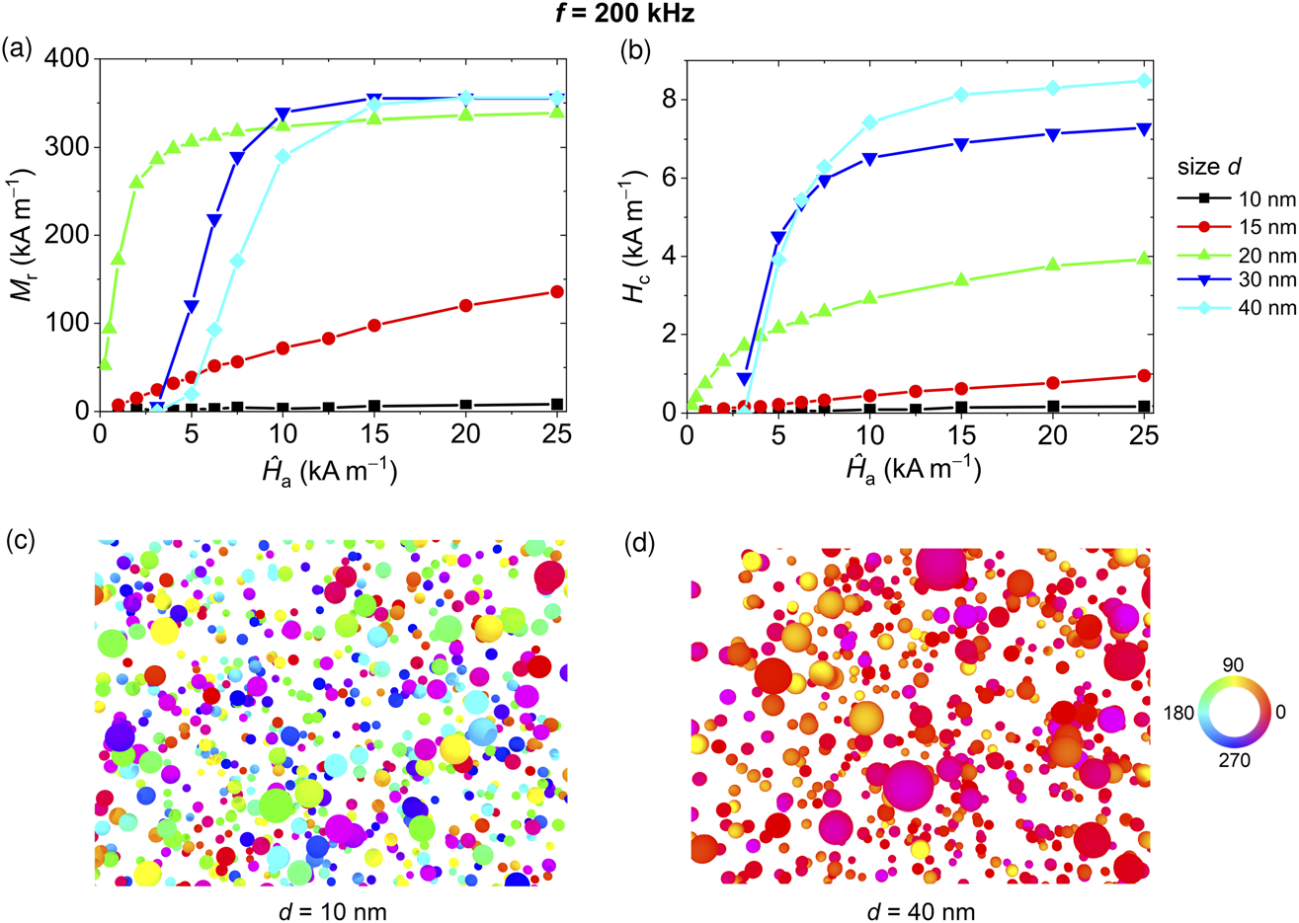
Influence of magnetic field peak amplitude *Ĥ*_a_ on (a) remanent magnetization and (b) coercivity of 200 kHz hysteresis loops of five samples of non-interacting Fe_3_O_4_ NPs with variable size *d* from 10 to 40 nm. Remanence states (along the descending branch) calculated for (c) *d* = 10 nm and (d) *d* = 40 nm when *Ĥ*_a_ = 20 kA m^−1^. The colours in the maps represent the angle (in degrees) between the magnetization and the positive direction of the applied magnetic field.

First, we calculate the dynamic hysteresis loops of samples composed of Fe_3_O_4_ NPs with variable diameter *d*, for different field peak amplitude *Ĥ*_a_ and frequency *f* fixed to 200 kHz (see Fig. S2 of the ESI†). The corresponding values of the remanent magnetization *M*_r_ and coercivity *H*_c_ are reported in Fig. 1a and b, respectively.

For *d* = 10 nm, *M*_r_ and *H*_c_ are practically negligible in all the ranges of the explored parameters, suggesting a superparamagnetic behaviour, as also demonstrated by the extremely small area of the calculated hysteresis loops (Fig. S2a†). Superparamagnetism is clearly visible also in Fig. 1c, which illustrates the remanence state for a sample of 10 nm NPs, obtained for a minor loop with *Ĥ*_a_ = 20 kA m^−1^. The magnetization is indeed randomly oriented with a net magnetic moment close to zero.

For *d* = 15 nm, the first hysteresis loops with non-negligible area appear when *Ĥ*_a_ is greater than 5 kA m^−1^ (Fig. S2b†). Then, both *M*_r_ and *H*_c_ increase with *Ĥ*_a_, reaching respectively 140 kA m^−1^ (0.35 *M*_S_) and 1 kA m^−1^, when *Ĥ*_a_ = 25 kA m^−1^. The observed behaviour indicates that the 15 nm sized NPs are in the transition state between reversible (superparamagnetic) and non-reversible (blocked) states.^62^

For *d* = 20 nm, the Fe_3_O_4_ NPs are characterized by minor loops with significant areas and non-negligible remanent magnetization and coercivity values, already starting from very low values of *Ĥ*_a_ (Fig. S2c†). Specifically, *M*_r_ shows an abrupt increase from 50 to 300 kA m^−1^ as *Ĥ*_a_ increases up to 4 kA m^−1^. Then, *M*_r_ converges towards a value, 340 kA m^−1^, which is around 0.83 *M*_S_. The shapes of the hysteresis loops suggest a transition state also for the 20 nm NPs.^63^

For *d* = 30 nm, the hysteresis loops have a square shape and the ones calculated for *Ĥ*_a_ lower than 3 kA m^−1^ have a very small area; an abrupt increase in the loop area appears when *Ĥ*_a_ passes from 3 to 10 kA m^−1^. In detail, *M*_r_ and *H*_c_ are practically negligible when *Ĥ*_a_ <3 kA m^−1^. For values of *Ĥ*_a_ between 3 and 10 kA m^−1^, *M*_r_ increases rapidly to nearly 0.85 *M*_S_, then reaching a plateau. The plateau is very close to the maximum remanent magnetization value (0.866 *M*_S_) predicted in the absence of thermal effects for a randomly oriented ensemble of non-interacting single-domain NPs with cubic anisotropy.^64^ This is a proof of the limited influence of the thermal contribution for this size range. *H*_c_ shows a similar rapid growth for values of *Ĥ*_a_ between 3 and 10 kA m^−1^; above this range, the coercivity slowly increases with the field with an almost linear behaviour. The obtained results demonstrate that the 30 nm Fe_3_O_4_ NPs have properties typical of magnetic NPs in the blocked state.

For *d* = 40 nm, *M*_r_ varies with *Ĥ*_a_ similarly to the case with *d* = 30 nm, apart from a shift in such behaviour of 2 kA m^−1^ towards higher values of *Ĥ*_a_. The coercivity increases in a similar way to *d* = 30 nm, but reaches an approximately 20% higher value when *Ĥ*_a_ is greater than 7.5 kA m^−1^, due to an even lower sensitivity to thermal noise associated with size increase. Hysteresis loops very close to the major one can be already obtained for *Ĥ*_a_ = 15 kA m^−1^, with very high remanent magnetization values. This is well depicted in Fig. 1d, which shows the remanence state along the descending branch for *Ĥ*_a_ = 20 kA m^−1^; the magnetization vector is practically aligned for nearly all the NPs.

**Fig. 2:**
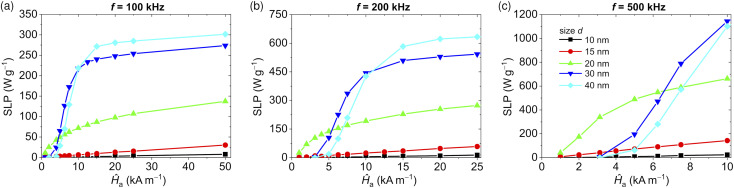
Influence of magnetic field peak amplitude *Ĥ*_a_ on the specific loss power (SLP) of five samples of non-interacting Fe_3_O_4_ NPs with variable size *d* from 10 to 40 nm, and fixing frequency *f* to (a) 100 kHz, (b) 200 kHz and (c) 500 kHz. In each graph, the maximum value of *Ĥ*_a_ is varied in accordance with the Hergt–Dutz limit.

The SLP values *versus Ĥ*_a_ are reported in Fig. 2 for the previously considered frequency of 200 kHz and, additionally, for *f* equal to 100 and 500 kHz. The smallest NPs (10–15 nm) are characterized by a linear increase in SLP within all the considered ranges of *Ĥ*_a_, but overall their heating efficiency is far from the one of large NPs. As an example, when *d* = 15 nm, the maximum SLP values reached within the Hergt–Dutz limit range are around 30, 60, and 140 W g^−1^ for *f* = 100, 200, and 500 kHz, respectively. Much greater values of SLP can be obtained with the 20 nm NPs, resulting in around 140, 280, and 660 W g^−1^ for *f* = 100, 200, and 500 kHz, respectively.

Apart from very low field amplitudes, the largest Fe_3_O_4_ NPs (30–40 nm) show the highest SLP values, which are characterized, however, by a nearly asymptotic behavior towards a maximum value, which depends on *d* and *f*. As an example, when *d* = 40 nm and *f* = 100 kHz, above 15 kA m^−1^ the SLP grows very slowly with the field, increasing by a very small percentage from 20 kA m^−1^ to 50 kA m^−1^ (see Fig. 2a). This result suggests that at 100 kHz, it is more convenient to activate 40 nm Fe_3_O_4_ NPs with AC magnetic fields with *Ĥ*_a_ around 15 kA m^−1^, also to limit possible undesired effects due to eddy current generation.^13^ Moreover, since the SLP directly scales with the frequency, it can be much better to limit *Ĥ*_a_ in favour of higher frequencies. Within the Hergt–Dutz limit range, when *d* = 40 nm and *f* = 200 kHz, the SLP reaches a 645 W g^−1^ plateau, which is more than double the value obtainable with a frequency of 100 kHz.

The different behaviour of the SLP *versus d*, *Ĥ*_a_ and *f* displayed in Fig. 2 can be explained in terms of the transition from superparamagnetic to the blocked state^62^ and the role of the thermal and magnetocrystalline anisotropy energies.^64,65^ When *d* is equal to 10 and 15 nm, for all the considered frequencies the thermal contribution is dominant and the very small increment of SLP is mainly caused by the rate of change of the applied magnetic field. As the size increases, the contribution of the thermal field reduces and the effects of magnetocrystalline anisotropy begin to appear, leading to square hysteresis loops. Above 20 nm the anisotropy field dominates the reversal process.

For the largest NPs (*d* equal to 30 and 40 nm), the thermal agitation is not enough to overcome the anisotropy energy barrier without a contribution sufficiently high from the Zeeman energy. Therefore, when *Ĥ*_a_ is much lower than the major loop coercivity H*, which can be roughly estimated with analytical formulas,^64,65^ the magnetic moment is negligible and the SLP tends to zero. As *Ĥ*_a_ approaches H*, there is an increase in the fraction of NPs able to overcome the anisotropy energy barrier and aligned with the most favourable anisotropy directions, with a steep rise in both *M*_r_ and *H*_c_, and thus in the SLP values. These effects are responsible for the sigmoid growth curves of the SLP *versus Ĥ*_a_ depicted in Fig. 2 for *d* equal to 30 and 40 nm, where the second inflection point appears when *Ĥ*_a_ overcomes H*.^64,65^

**Fig. 3:**
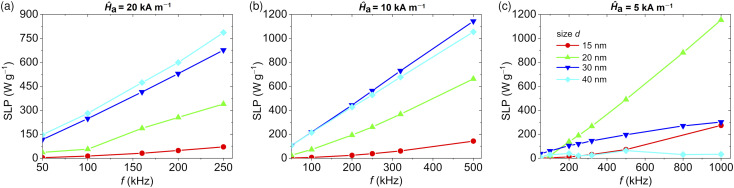
Influence of magnetic field frequency *f* on the SLP of four samples of non-interacting Fe_3_O_4_ NPs with variable size *d* from 15 to 40 nm, and fixing magnetic field peak amplitude *Ĥ*_a_ to (a) 20 kA m^−1^, (b) 10 kA m^−1^ and (c) 5 kA m^−1^. In each graph, the maximum value of *f* is varied in accordance with the Hergt–Dutz limit.

To better highlight the role of frequency, Fig. 3 shows the SLP as a function of *f* for three different values of *Ĥ*_a_, namely 20, 10 and 5 kA m^−1^, considering Fe_3_O_4_ NPs with sizes from 15 to 40 nm (when *d* = 10 nm, the SLP is practically negligible). The relative graphs of *M*_r_ and *H*_c_ are reported in Fig. S3 and S4 of the ESI,† respectively. For *Ĥ*_a_ = 20 kA m^−1^ (Fig. 3a), the SLP steadily increases with *f* up to the limit of 250 kHz, thanks to a gradual widening of the hysteresis loops observed for all the analyzed sizes. In this range of field parameters, the most efficient NPs are the largest ones.

For *Ĥ*_a_ = 10 kA m^−1^ (Fig. 3b), an increase in *f* up to 500 kHz corresponds again to an increase in SLP for all the considered sizes, despite the reduction in *M*_r_ for NPs with *d* ≥30 nm. For this range of field parameters, the 30 nm sized NPs are the most efficient ones.

For *Ĥ*_a_ = 5 kA m^−1^ (Fig. 3c), the increase in *f* up to 1 MHz is associated with a strong rise in SLP for the 20 nm NPs, reaching a value of ∼1150 W g^−1^, while the largest ones (*d* = 40 nm) are in a condition below the major loop coercivity H*, thus exhibiting a very low SLP. In this case, only a fraction of NPs, *i.e.* the ones with an easy direction parallel or almost parallel to the AC magnetic field, experiences an irreversible transition between the anisotropy energy minima. This transition can be thermally activated, therefore, the switching probability is temperature- and time-dependent. As *f* increases, the probability of NPs overcoming the anisotropy energy barrier reduces, with a consequent decrease in the ensemble magnetic moment along the applied field direction, causing the hysteresis loop contraction. For this reason, large NPs show a sublinear increase in the SLP *versus f* (Fig. 3c). On the other hand, in the low field range, for the NPs with smaller diameters (*d* equal to 15 and 20 nm), an increase in *f* corresponds to an increment of both *M*_r_ (Fig. S3 of the ESI†) and *H*_c_ (Fig. S4 of the ESI†). For this kind of NP, the use of fields with low *Ĥ*_a_ and high *f* is a viable strategy to amplify the heat generation.

To explore the role of size in much more detail, we perform additional simulations gradually changing the NP diameter between 10 and 40 nm, under the following excitation conditions that fulfil the Hergt–Dutz limit:#1 – *Ĥ*_a_ = 20 kA m^−1^, *f* = 250 kHz;#2 – *Ĥ*_a_ = 10 kA m^−1^, *f* = 500 kHz;#3 – *Ĥ*_a_ = 5 kA m^−1^, *f* = 1 MHz.

**Fig. 4:**
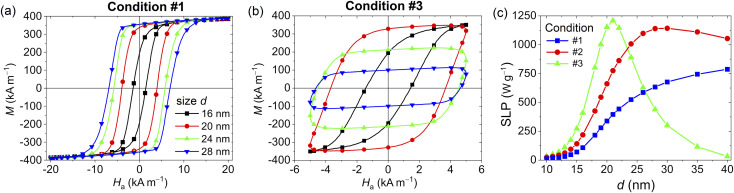
Dynamic hysteresis loops of four samples of non-interacting Fe_3_O_4_ NPs with variable size *d* within a restricted range (16–28 nm), calculated under excitation conditions (a) #1 – *Ĥ*_a_ = 20 kA m^−1^, *f* = 250 kHz and (b) #3 – *Ĥ*_a_ = 5 kA m^−1^, *f* = 1 MHz. (c) SLP as a function of NP size in the range 10–40 nm, calculated for samples of non-interacting NPs under excitation conditions #1, #3 and #2 – *Ĥ*_a_ = 10 kA m^−1^, *f* = 500 kHz.

Fig. 4a and b show the hysteresis loops calculated for conditions #1 and #3, respectively, focusing on a narrow size interval (16–28 nm), while Fig. 4c reports the SLP values for all the analysed conditions. For the highest field/lowest frequency case (condition #1), the loops enlarge gradually with *d*, tending to become square; this leads to a monotonic behaviour *versus d* of the SLP. For the lowest field/highest frequency case (condition #3), the loop shape varies significantly with *d*, with the area initially growing and then progressively reducing. As a consequence, the SLP has no more a monotonic behaviour with NP size,^63^ showing a peak of ∼1210 W g^−1^ when *d* = 21 nm. A similar but less pronounced trend is observed also for the excitation condition #2, where the SLP peak (∼1140 W g^−1^) is around 30 nm. Below the SLP peak sizes, the thermal noise enables an easier switching between anisotropy energy minima, leading to lower values of *M*_r_ and *H*_c_. Above those sizes, the decrease in the thermal energy contribution reduces the probability of the thermally activated switching. The magnetization reversal becomes therefore mainly governed by the relative orientation of the NP magnetocrystalline anisotropy axes with respect to the applied field.

It is clear from Fig. 4c that the heating properties of Fe_3_O_4_ NPs can change drastically depending on the field parameters, requiring a careful selection of the excitation conditions during *in vivo* treatments.^66^ As an example, when *d* = 40 nm an SLP of ∼1050 W g^−1^ and ∼790 W g^−1^ can be reached in cases #2 and #1, respectively, while it becomes practically negligible in case #3 for that size. When *d* = 20 nm, from case #3 to #1 the SLP reduces to approximately one third, passing from ∼1150 W g^−1^ to ∼340 W g^−1^.

The behaviour of the SLP *versus d*, *Ĥ*_a_ and *f* shown in Fig. 2, 3 and 4c is qualitatively similar to the one observed in previous experimental studies of magnetic NPs with size below multidomain transition.^20,67–69^ Quantitative discrepancies between the results are associable with different material composition, non-spherical shape, strong dependence of material properties on the used chemical synthesis process, surface anisotropy effects, aggregate formation, *etc.* Moreover, it is worth noting that the estimation of SLP from thermometric and calorimetric measurements can suffer from uncertainties and errors imposed by both experimental conditions and the estimation methodology.^70^ This limits the reproducibility and accuracy of SLP values, and makes the direct comparison with simulation results critical, unless analytical fitting models are used.

Finally, it is noteworthy that in a real ensemble, the NPs are not perfect spheres, due to unavoidable defects coming from the synthesis process. As a consequence, weak shape anisotropies can appear, with a possible impact on the SLP values. Anyway, if the variation in the NP aspect ratio is limited, the heating properties are weakly affected, as shown in Fig. S5 of the ESI,† for the different NP sizes here considered.

### 3.2. Interacting magnetic nanoparticles: role of volume concentration and aggregation order

Magnetostatic dipole–dipole interactions can have a strong influence on the shape and area of magnetic NP hysteresis loops, thus affecting the NP heating properties.^71,72^ Here, we study how the SLP of an ensemble of Fe_3_O_4_ NPs varies as a function of aggregation order and volume concentration *σ*. Parameter *σ* is defined as the ratio *V*_NPs_/*V*_system_, where *V*_NPs_ is the volume of one NP multiplied by the number of NPs belonging to the ensemble, and *V*_system_ is the volume of the smallest box containing all the NPs.

**Fig. 5:**
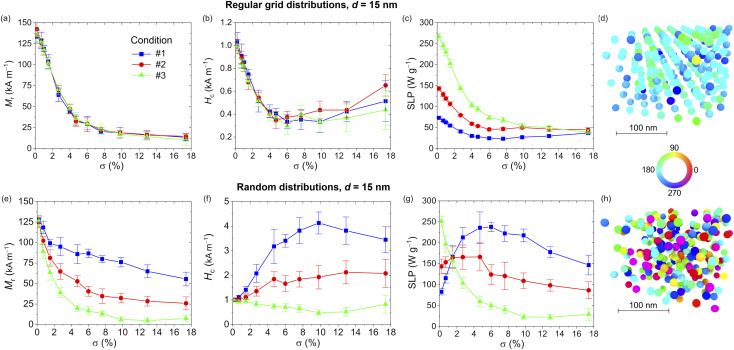
Influence of volume concentration *σ* for interacting 15 nm sized Fe_3_O_4_ NPs distributed on 3D regular grids: (a) remanent magnetization; (b) coercivity; (c) SLP. Influence of volume concentration *σ* for interacting 15 nm sized Fe_3_O_4_ NPs randomly distributed in the space: (e) remanent magnetization; (f) coercivity; (g) SLP. The data correspond to the average of eight realizations with the bars representing the standard deviation, and are obtained under three excitation conditions: #1 – *Ĥ*_a_ = 20 kA m^−1^, *f* = 250 kHz; #2 – *Ĥ*_a_ = 10 kA m^−1^, *f* = 500 kHz; #3 – *Ĥ*_a_ = 5 kA m^−1^, *f* = 1 MHz. Magnetization configurations calculated for *σ* = 6%, setting excitation condition at #1 and field step at −3 kA m^−1^: comparison between (d) regular grid and (h) random arrangements. The colours in the maps represent the angle (in degrees) between the magnetization and the positive direction of the applied magnetic field.

**Fig. 6:**
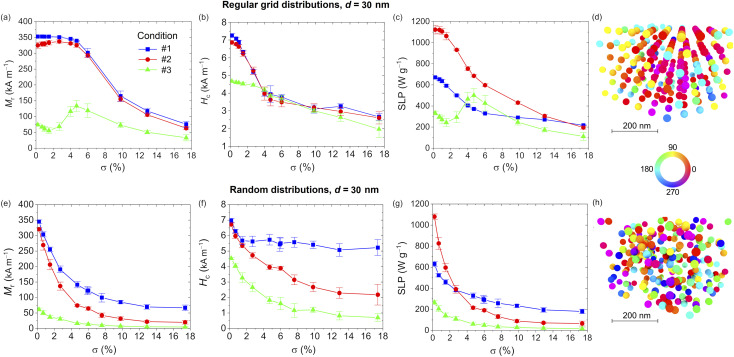
Influence of volume concentration *σ* for interacting 30 nm sized Fe_3_O_4_ NPs distributed on 3D regular grids: (a) remanent magnetization; (b) coercivity; (c) SLP. Influence of volume concentration *σ* for interacting 30 nm sized Fe_3_O_4_ NPs randomly distributed in the space: (e) remanent magnetization; (f) coercivity; (g) SLP. The data correspond to the average of eight realizations with the bars representing the standard deviation, and are obtained under three excitation conditions: #1 – *Ĥ*_a_ = 20 kA m^−1^, *f* = 250 kHz; #2 – *Ĥ*_a_ = 10 kA m^−1^, *f* = 500 kHz; #3 – *Ĥ*_a_ = 5 kA m^−1^, *f* = 1 MHz. Magnetization configurations calculated at the remanence state for *σ* = 6%, setting excitation condition at #3: comparison between (d) regular grid and (h) random arrangements. The colours in the maps represent the angle (in degrees) between the magnetization and the positive direction of the applied magnetic field.

The role of *σ* is illustrated in Fig. 5 and 6, for *d* equal to 15 and 30 nm, respectively. The data are calculated for the three excitation conditions previously considered. In both figures the effect of the aggregation order is also investigated, comparing *M*_r_, *H*_c_ and SLP evaluated for the NPs distributed on 3D regular grids with hexagonal closed packing (top) and randomly in the space (bottom). The values reported in the graphs correspond to the average of eight realizations with the bars representing the standard deviation; for each realization, ensembles of 250 NPs are considered.

The analysis starts focusing on the Fe_3_O_4_ NPs with *d* = 15 nm and distributed on regular grids with *σ* varying between 0 and 17.4%; the relative hysteresis loops are shown in Fig. S6 of the ESI.† For all three excitation conditions, with the increase in *σ* the loop branches become more tilted, with a progressive reduction in the remanent magnetization (Fig. 5a). Conversely, *H*_c_ (Fig. 5b) has a non-monotonic behavior with a strong decrease up to *σ* ∼6%, followed by a slow rise due to the appearance of dipolar anisotropy effects. The lower distance between NPs is indeed responsible for the creation of easy directions for the magnetization orientation.

The effect of magnetostatic dipole–dipole interactions becomes appreciable on the SLP from center-to-center distances lower than 5*d*, *i.e.* for *σ* >0.7% (Fig. 5c). In the range of variation of *σ*, such an effect leads to a significant decrement of the SLP in comparison with the cases on non-interacting NPs (*σ* = 0), especially for excitation condition #3. For very large volume concentrations (*σ* > 15%), the SLP results in lower than 50 W g^−1^ for all the considered excitation conditions, with a reduction, with respect to the cases of infinite dilution, of 85%, 70% and 50% for conditions #3, #2 and #1, respectively.

Also when introducing stochasticity in the NP spatial distribution, the hysteresis loops (Fig. S7 of ESI†) tend to become more tilted as *σ* increases, with a monotonic decrease of the remanent magnetization for all the excitation conditions (Fig. 5e). The coercivity is more influenced by changes of *σ* for condition #1, exhibiting a non-monotonic behavior with a peak around a volume concentration of 10% (Fig. 5f). For the other two excitation conditions lower variations in *H*_c_ are observed. The combined effects on *M*_r_ and *H*_c_ are reflected in the behavior of the SLP (Fig. 5g); for excitation condition #1, being the most efficient one at infinite dilution, this is mainly governed by the reduction of *M*_r_, with a decrease similar to the case of NPs arranged on regular grids. For the other two excitation conditions, a non-monotonic behavior is obtained. As an example, for condition #1, the SLP is maximized when *σ* is around 6% reaching a value in the order of 240 W g^−1^. This behavior is strongly different from the one found with the NPs arranged on regular grids, for which under the same operative conditions the SLP is one order of magnitude lower.

The diverse reversal processes are well evident by comparing the magnetization configurations calculated for *σ* = 6% and a field step of −3 kA m^−1^, considering excitation condition #1. In particular, for the regular grid arrangement, most of the NPs have already switched their magnetization, tending to saturation (Fig. 5d). Conversely, for the random arrangement, there is a large variety of magnetization orientations, with a prevalence of NPs not having started the reversal process yet (Fig. 5h).

The analysis proceeds to focus on the Fe_3_O_4_ NPs with *d* = 30 nm and distributed on regular grids with *σ* varying between 0 and 17.4%; the relative hysteresis loops are shown in Fig. S8 of the ESI.† Depending on the excitation conditions, two very different behaviors appear, as already observed for the non-interacting NP cases. The first behavior, characteristic of low amplitude/ high frequency fields (condition #3), is mainly driven by the anisotropy energy, of both magnetocrystalline and dipolar origin, the latter due to the formation of preferential paths for the magnetization along the grid axes.

The second behavior, occurring at large fields, is common for the other excitation conditions, characterized by an important contribution from Zeeman energy. For excitation condition #3, *M*_r_ presents a peak around a volume concentration of 5% (Fig. 6a), while *H*_c_ diminishes linearly as *σ* increases (Fig. 6b); as a result, the SLP has a non-monotonic behavior with a maximum of 500 W g^−1^ for *σ* = 4.7% (Fig. 6c). The high remanent magnetization found at this value of *σ* is well evident in the map of Fig. 6d, where large arrays of contiguous NPs with magnetization pointing in the direction of the previous maximum field state can be distinguished.

For excitation conditions #2 and #3, *M*_r_ is weakly influenced by magnetostatic dipole–dipole interactions for low values of *σ* (up to 5%), and then a gradual decrease follows (Fig. 6a). Conversely, *H*_c_ diminishes more rapidly up to *σ* = 5%, with a successive very small decrement (Fig. 6b). As a consequence, the increase in *σ* leads to a reduction of the SLP, more pronounced in the low *σ* range and for the most efficient excitation condition (#2), with SLP varying from 1140 W g^−1^ for infinite dilution to around 200 W g^−1^ for *σ* >15%.

As can be observed in Fig. S9,† when introducing stochasticity in the NP spatial distribution, a continuous reduction of the hysteresis loop area *versus σ* is obtained for all three excitation conditions, associated with a decrease in both *M*_r_ (Fig. 6e) and *H*_c_ (Fig. 6f). This leads to a progressive decay of the SLP (Fig. 6g), resulting to be more pronounced for the excitation condition #2, as for the case of NPs distributed on regular grids. The non-monotonic behavior of SLP observed for condition #3 and ordered NP ensembles is no more present, due to the minor contribution of dipolar anisotropy effects. The reduction of the influence of such effects is confirmed by the remanence state calculated for condition #3 setting *σ* at 4.7% (Fig. 6h), where a great level of disorder can be observed, differently from what happens for the regular grid distribution at the same volume concentration (Fig. 6d).

A diverse behavior between the two types of NP arrangements is well evident also at very low values of *σ*. Specifically, for the regular grid distributions the magnetostatic dipole–dipole interactions are negligible up to *σ* of 1%, corresponding to center-to-center distances higher than 4.5*d*. In this interval of *σ*, the SLP is characterized by small variations, differently from the stochastic distribution cases, where significant decreases can be observed starting from *σ* of 0.7%. A reduction in the order of 20% is indeed found when passing from infinite dilution to *σ* = 0.7% under excitation condition #2. This behavior can be ascribed to the presence of locally denser NP clusters with an average nearest neighbor distance of around 3.5*d*. In these zones of the aggregates, the magnetostatic dipole–dipole interactions are stronger and produce an effect impacting the overall hysteresis loop.

## 4 Conclusions

This study has explored, with a micromagnetic modelling approach, 10–40 nm sized Fe_3_O_4_ NPs for magnetic hyperthermia application. Modelling has proved to be a valid tool for supporting nanomaterial engineers in finding the optimal combination of NP size and field parameters (frequency *f* and peak amplitude *Ĥ*_a_) to maximize heating properties while fulfilling conditions compatible with application on living beings.

For very diluted concentrations, when varying the frequency between 50 kHz and 1 MHz, the best heating performances can be obtained with 20 nm NPs excited by a field with *f* = 1 MHz and *Ĥ*_a_ = 5 kA m^−1^, or with 30 nm NPs excited by a field with *f* = 500 kHz and *Ĥ*_a_ = 10 kA m^−1^. In these cases, SLP values higher than 1100 W g^−1^ can be reached. For larger field peak amplitudes and lower frequencies, the optimal NPs are the 40 nm ones, but the achievable SLP values reduce, *e.g.* with *f* = 100 kHz and *Ĥ*_a_ = 50 kA m^−1^, SLP around 300 W g^−1^ can be obtained.

For denser NP aggregates, the magnetostatic dipole–dipole interactions influence the hysteresis losses of an amount that depends on the NP arrangement. The behavior of regular grid distributions strongly differs from the one of randomly clustered arrangements, making non reliable the approximation with equally spaced NPs, apart from very low volume concentrations. Focusing on stochastic distributions, the geometrical parameter mainly influencing the SLP is not the average volume concentration, but the average nearest neighbor distance between NPs belonging to smaller sub-groups, where the magnetostatic interactions are locally amplified. The obtained results put in evidence how an accurate investigation of the level of dispersion and of the role of the aggregation order is required for the correct estimation of SLP under realistic hyperthermia conditions.

As a future direction, our micromagnetic modelling approach can provide inputs to *in silico* models implementing the bioheat transfer equation, in terms of the specific heating power released by NPs. Combining micromagnetic and thermal simulations can be a valid strategy for hyperthermia treatment planning, allowing the determination of the spatial-temporal distribution of the temperature in the target region, *versus* the magnetic NP heating properties, the AC magnetic field parameters, the duty cycle, the magnetic NP local concentration, as well as the position of the tumor within the body and its thermal properties.

Benefits are also expected from the use of micromagnetic-thermal modelling in synergy with imaging techniques, like MPI, which can accurately quantify magnetic NP concentration and spatial distribution within target tissues, providing feedback for the modulation of excitation conditions (*i.e.*, magnetic field duty cycle and amplitude). When internalized in cancer cells, the magnetic NPs can indeed strongly aggregate, therefore the quantification of magnetostatic dipole–dipole interactions and the evaluation of the influence on SLP of NP local concentration, derived from tomographic images, can play a pivotal role in treatment planning. Moreover, imaging techniques like MPI can be used to sample the tissue temperature at discrete time intervals, and thus to address power adjustment to ensure that energy delivery conforms to a prescribed treatment plan. This objective can be achieved only if the NP heating properties *versus* AC magnetic field parameters are known, as envisaged in our work. However, effective and reliable utilization of the computed data can be achieved only after a preliminary comparison with experimental data obtained on the NPs selected for the hyperthermia treatment. This will permit us to tune model inputs, simulating in a more realistic way the impact of material composition, dependence of material properties on the used chemical synthesis process, surface anisotropy effects, not perfect spherical shape, size dispersion, possible multicore structures, and aggregate formation (clusters and chains).

## Conflicts of interest

There are no conflicts to declare.

## Supplementary Material

NA-006-D3NA00709J-s001
