# Supplementary material for: Influence of size, volume concentration and aggregation state on magnetic nanoparticle hyperthermia properties versus excitation conditions
Source: Nanoscale Adv. 2024 Feb 14;6(6):1739–49. doi: 10.1039/d3na00709j (PMC10929599; doi:10.1039/d3na00709j)
Supplement: NA-006-D3NA00709J-s001 [file NA-006-D3NA00709J-s001.pdf]

Electronic Supplementary Material (ESI) for Nanoscale Advances.
This journal is © The Royal Society of Chemistry 2024

Electronic Supplementary Information

Influence of size, aggregation state and excitation conditions on hyperthermia properties of magnetite nanoparticles

Riccardo Ferrero,[a] Marta Vicentini [a] and Alessandra Manzin[a]

[a]Istituto Nazionale di Ricerca Metrologica (INRiM), Strada delle Cacce 91, 10135 Torino, Italy

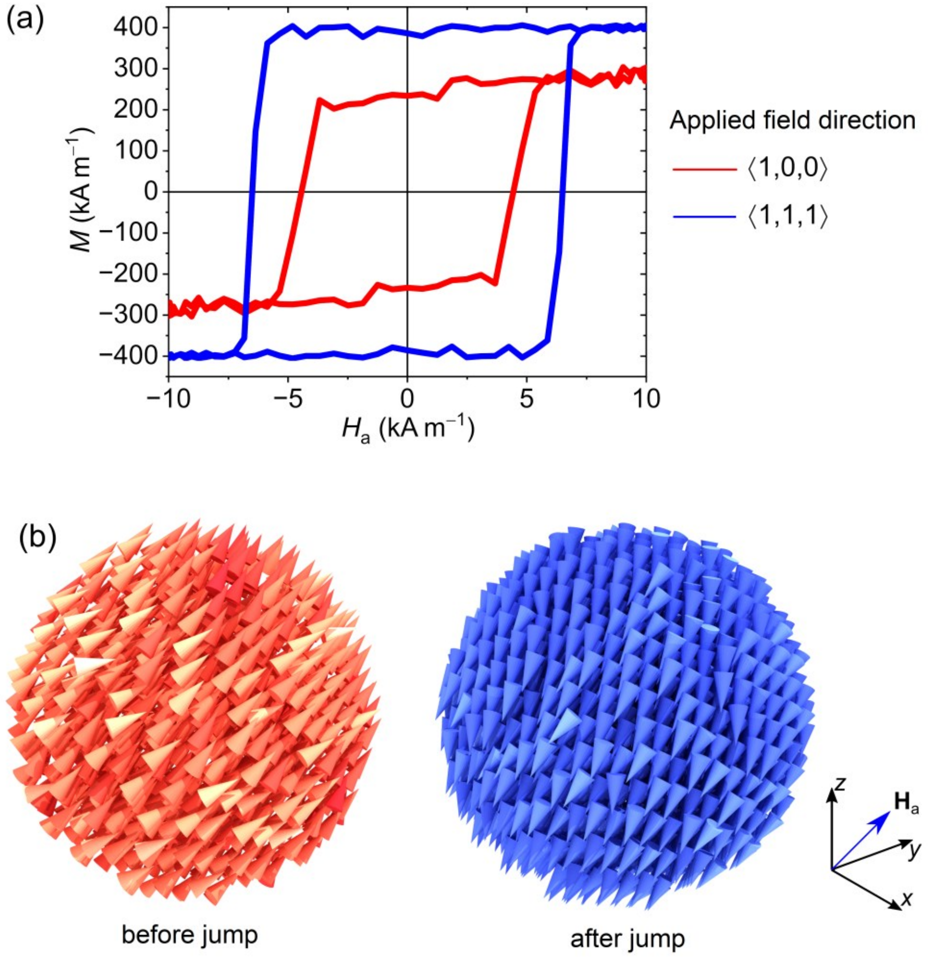

Fig. S1 3D micromagnetic simulation of a Fe_3O_4 nanosphere with 40 nm diameter, discretized with cubic cells of 2.5 nm size and having magnetocrystalline hard axes set along the Cartesian ones. (a) Comparison of the dynamic hysteresis loops calculated by applying a 100 kHz magnetic field with peak amplitude of 10 kA m^{-1} along the hard direction <1,0,0> and easy direction <1,1,1>. (b) Magnetization configurations at the time instants immediately before (left) and after (right) the irreversible jump along the descending branch of the hysteresis loop, calculated for the applied field direction <1,1,1>.

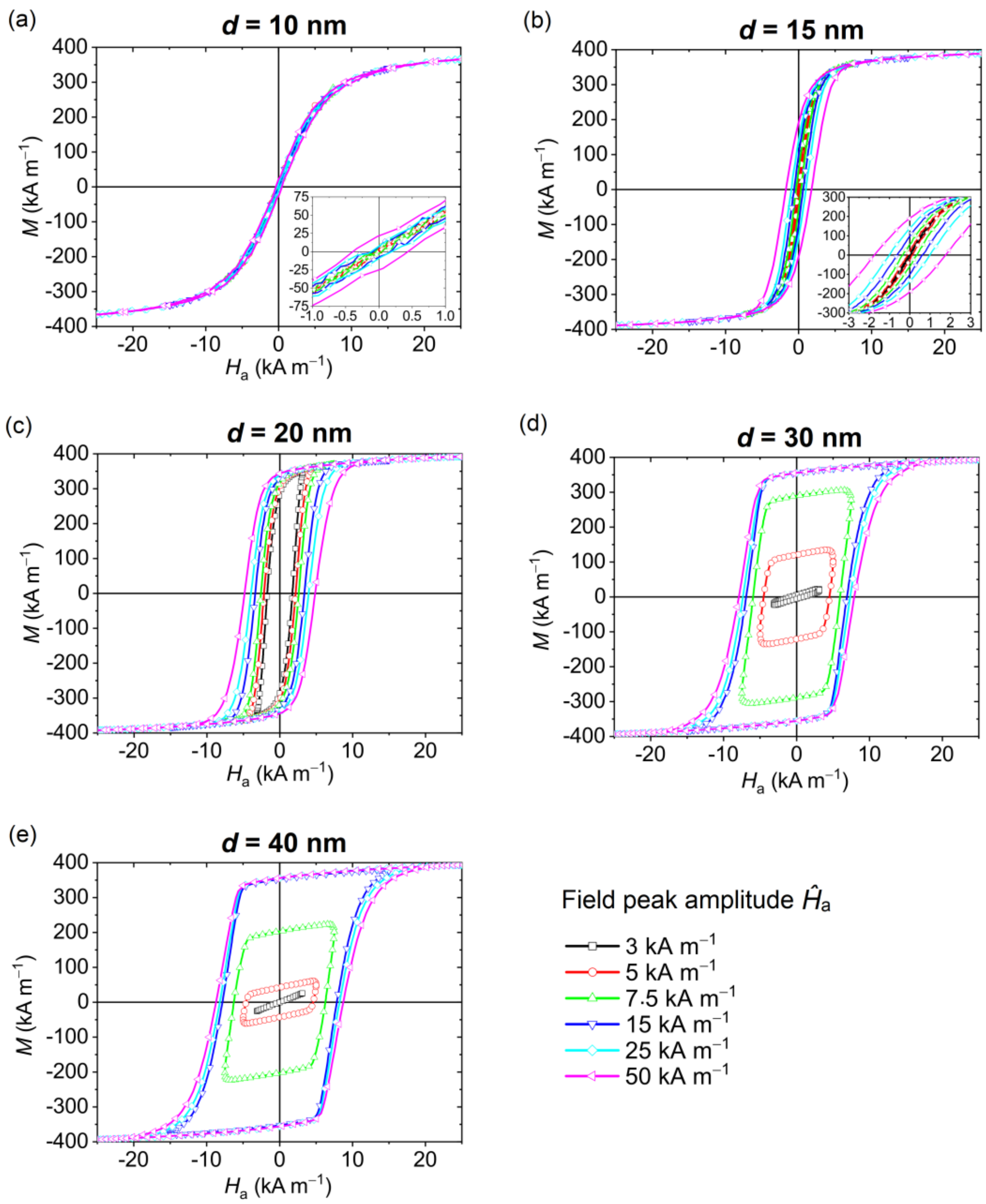

Fig. S2 Dynamic hysteresis loops calculated for five samples of non-interacting Fe_3O_4 NPs with size *d* fixed to (a) 10 nm, (b) 15 nm, (c) 20 nm, (d) 30 nm and (e) 40 nm. The frequency of the applied magnetic field is set at 200 kHz for all the simulations, and the peak amplitude $\hat{H}_a$ varies between 3 and 50 kA m^{-1}. Hergt-Dutz limit is fulfilled up to $\hat{H}_a$ = 25 kA m^{-1}.

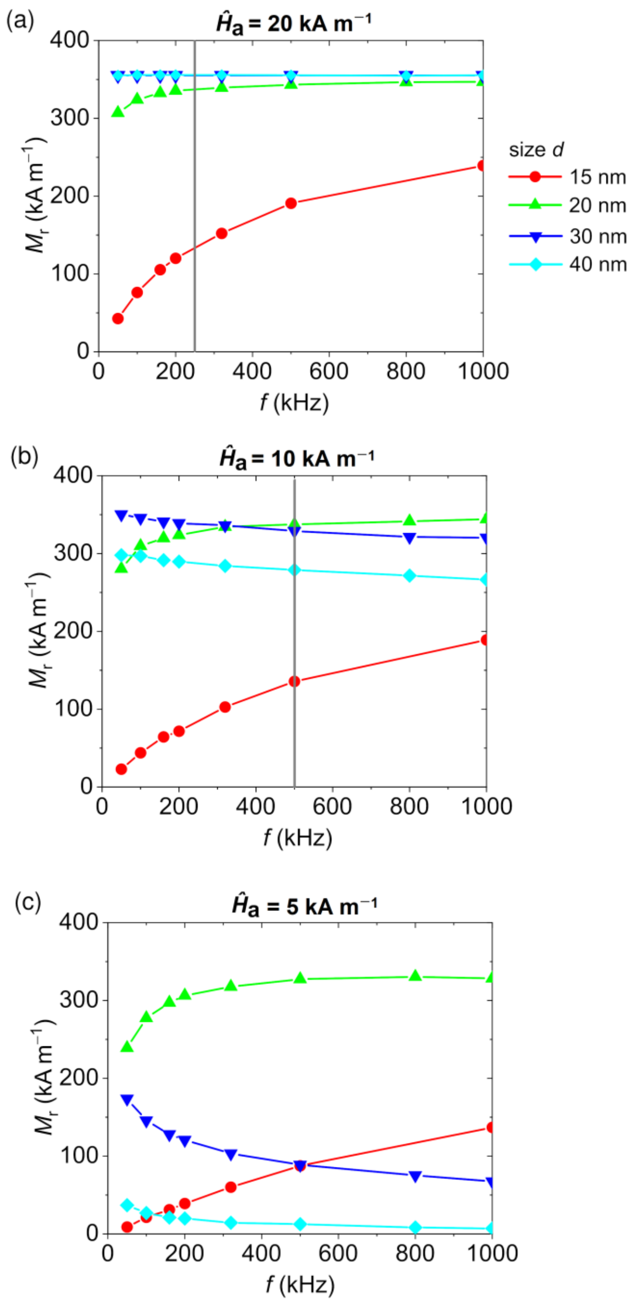

Fig. S3 Influence of AC magnetic field frequency *f* on the remanent magnetization of four samples of non-interacting Fe_3O_4 NPs with size *d* variable from 15 to 40 nm, fixing magnetic field peak amplitude $\hat{H}_a$ to (a) 20 kA m^{-1}, (b) $\hat{H}_a$ = 10 kA m^{-1} and (c) $\hat{H}_a$ = 5 kA m^{-1}. In graphs (a) and (b) the vertical grey lines represent the Hergt-Dutz limit.

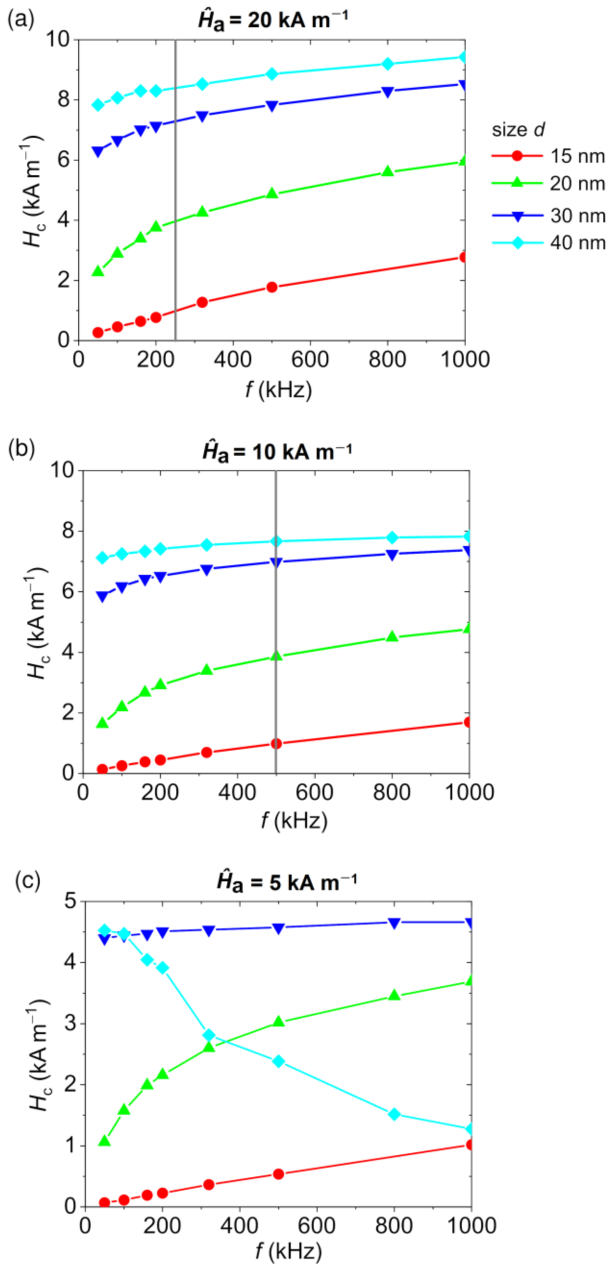

Fig. S4 Influence of AC magnetic field frequency *f* on the coercivity of four samples of non-interacting Fe_3O_4 NPs with size *d* variable from 15 to 40 nm, fixing magnetic field peak amplitude $\hat{H}_a$ to (a) 20 kA m^{-1}, (b) $\hat{H}_a$ = 10 kA m^{-1} and (c) $\hat{H}_a$ = 5 kA m^{-1}. In graphs (a) and (b) the vertical grey lines represent the Hergt-Dutz limit.

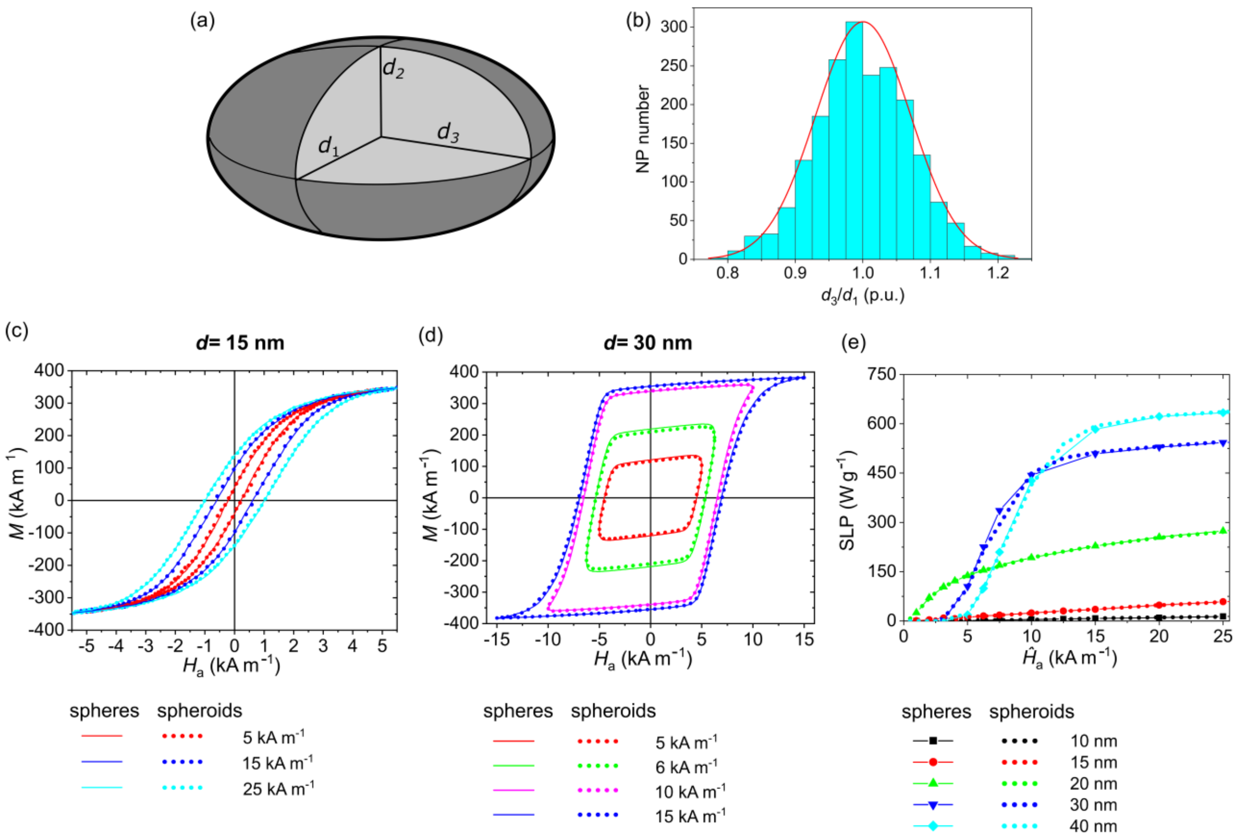

Fig. S5 Results for ensembles of 2000 infinitely diluted Fe_3O_4 NPs with randomly variable spheroidal shape around the spherical one. (a) Schematic of a spheroid, with aspect ratio d_3/d_1, where d_3 is the length along the spheroid symmetry axis and $d_1 = d_2$ is the equatorial diameter. (b) Considered Gaussian distribution of NP aspect ratios, with mean equal to 1 ($d_1 = d_2 = d_3 = d$), standard deviation equal to 0.07, and NP volume fixed to $\pi d^3/6$. Dynamic hysteresis loops calculated at 200 kHz for variable magnetic field peak amplitude, setting d at (c) 15 nm and (d) 30 nm: comparison between random distributions (dotted lines) and ensembles with perfectly spherical NPs (continous lines). (e) Specific loss power (SLP) calculated for d ranging from 10 to 40 nm: comparison between random distributions (dotted lines) and ensembles with perfectly spherical NPs (lines with markers).

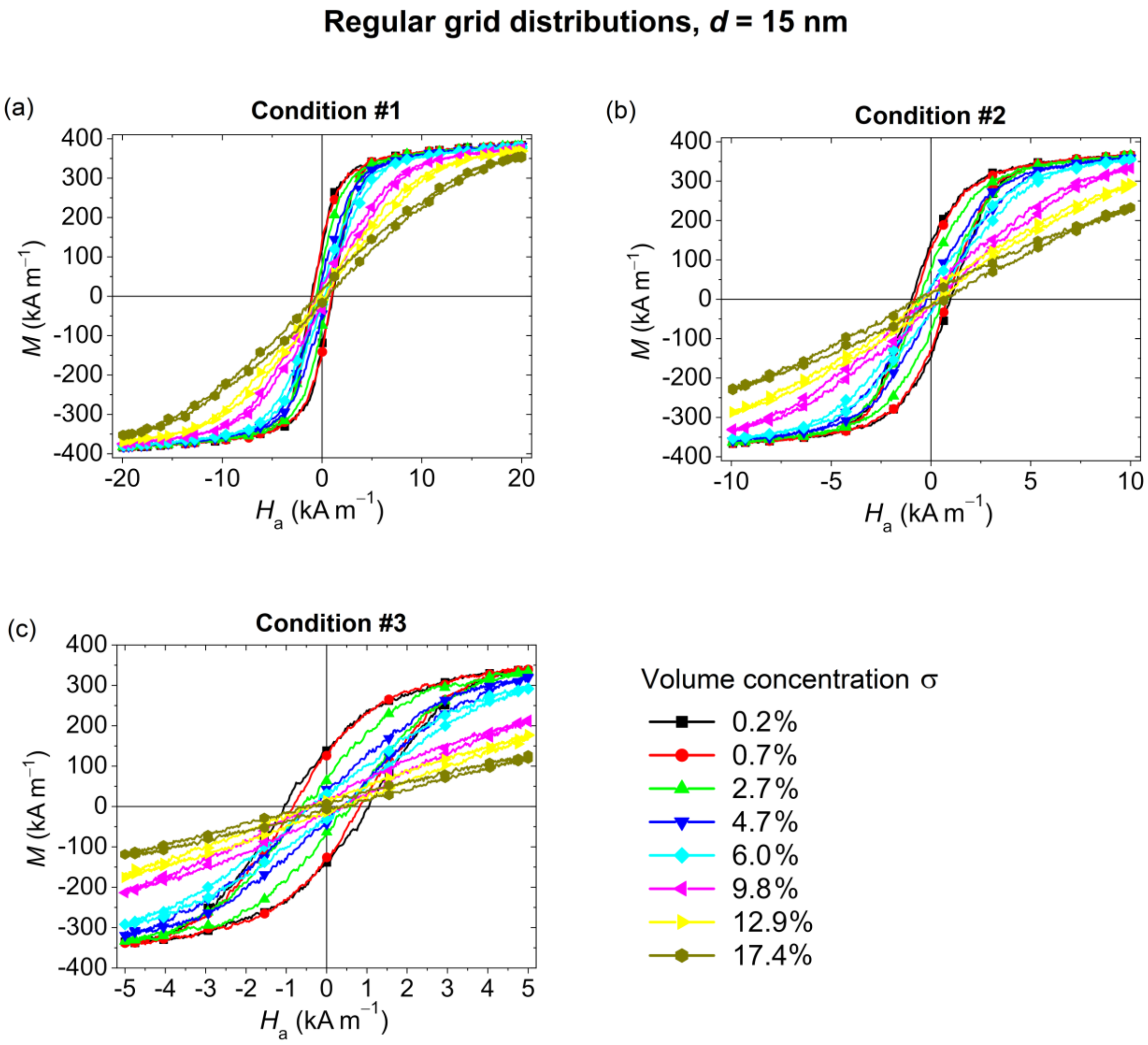

Fig. S6 Dynamic hysteresis loops versus volume concentration σ for interacting 15 nm sized Fe_3O_4 NPs distributed on 3D regular grids, calculated under three excitation conditions: #1 − $\hat{H}_a$ = 20 kA m^{-1}, f = 250 kHz; #2 − $\hat{H}_a$ = 10 kA m^{-1}, f = 500 kHz; #3 − $\hat{H}_a$ = 5 kA m^{-1}, f = 1 MHz.

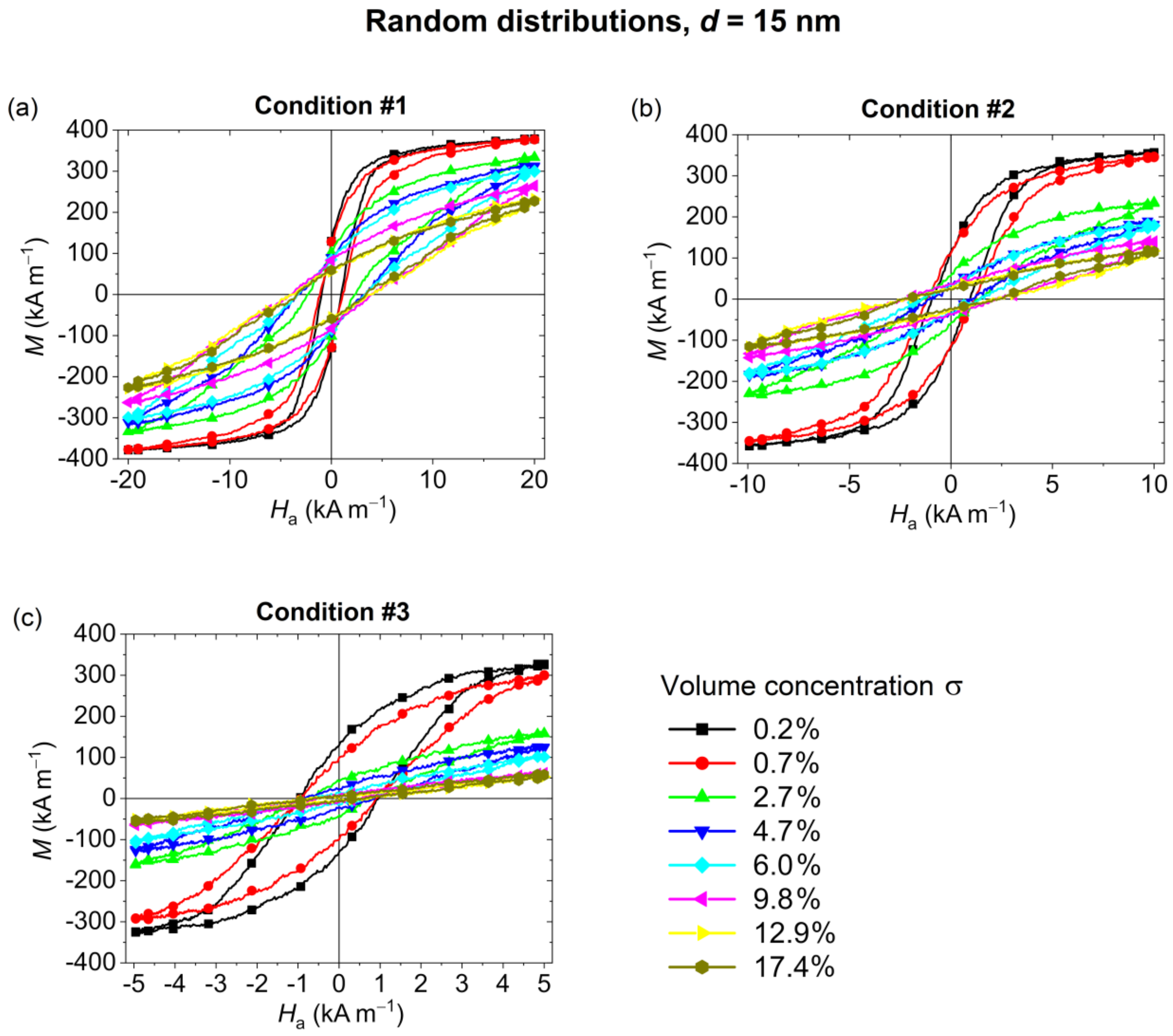

Fig. S7 Dynamic hysteresis loops versus volume concentration σ for interacting 15 nm sized Fe_3O_4 NPs randomly distributed in the space, calculated under three excitation conditions: #1 − $\hat{H}_a$ = 20 kA m^{-1}, f = 250 kHz; #2 − $\hat{H}_a$ = 10 kA m^{-1}, f = 500 kHz; #3 − $\hat{H}_a$ = 5 kA m^{-1}, f = 1 MHz.

Regular grid distributions, *d* = 30 nm

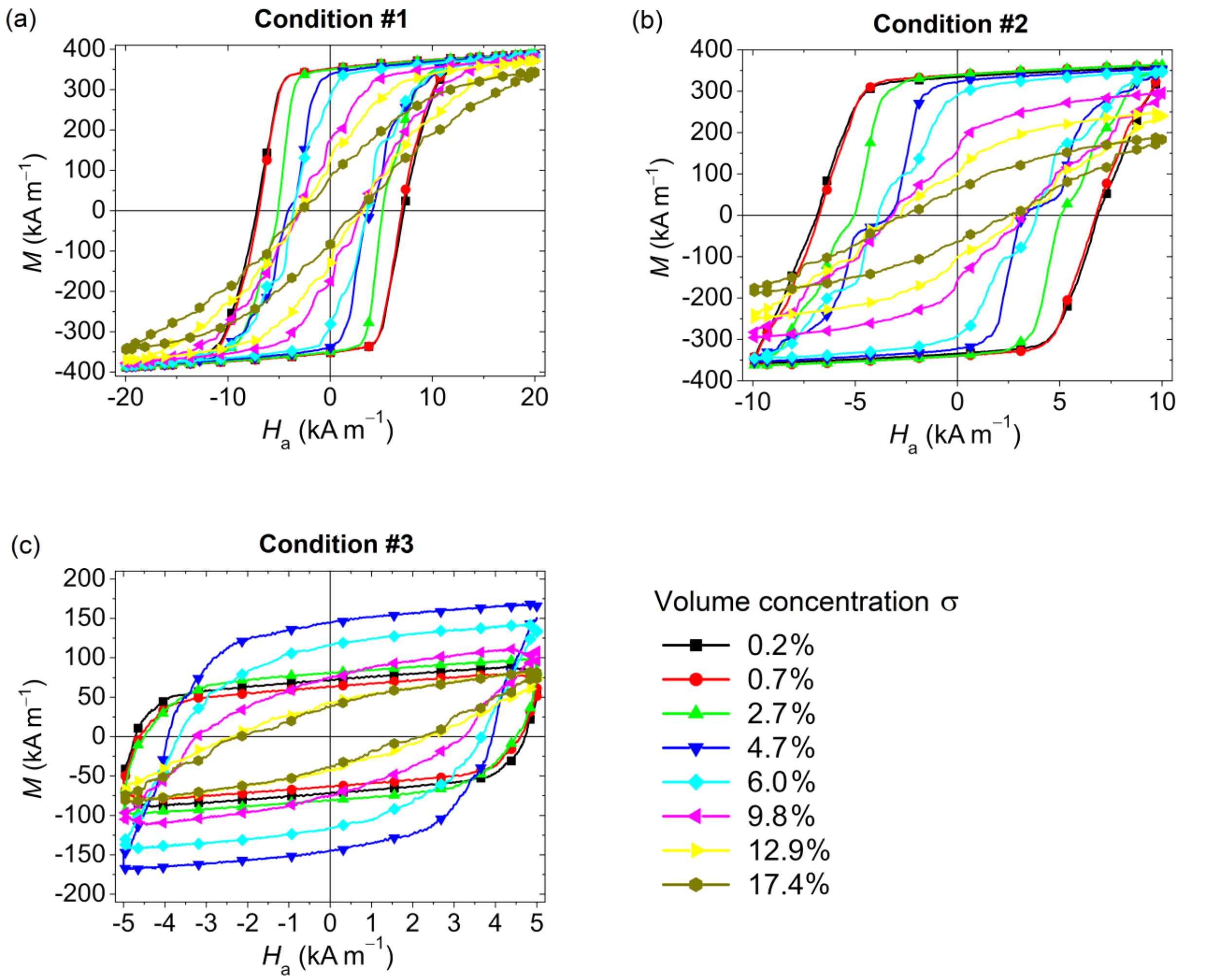

Fig. S8 Dynamic hysteresis loops versus volume concentration σ for interacting 30 nm sized Fe_3O_4 NPs distributed on 3D regular grids, calculated under three excitation conditions: #1 − $\hat{H}_a$ = 20 kA m^{-1}, f = 250 kHz; #2 − $\hat{H}_a$ = 10 kA m^{-1}, f = 500 kHz; #3 − $\hat{H}_a$ = 5 kA m^{-1}, f = 1 MHz.

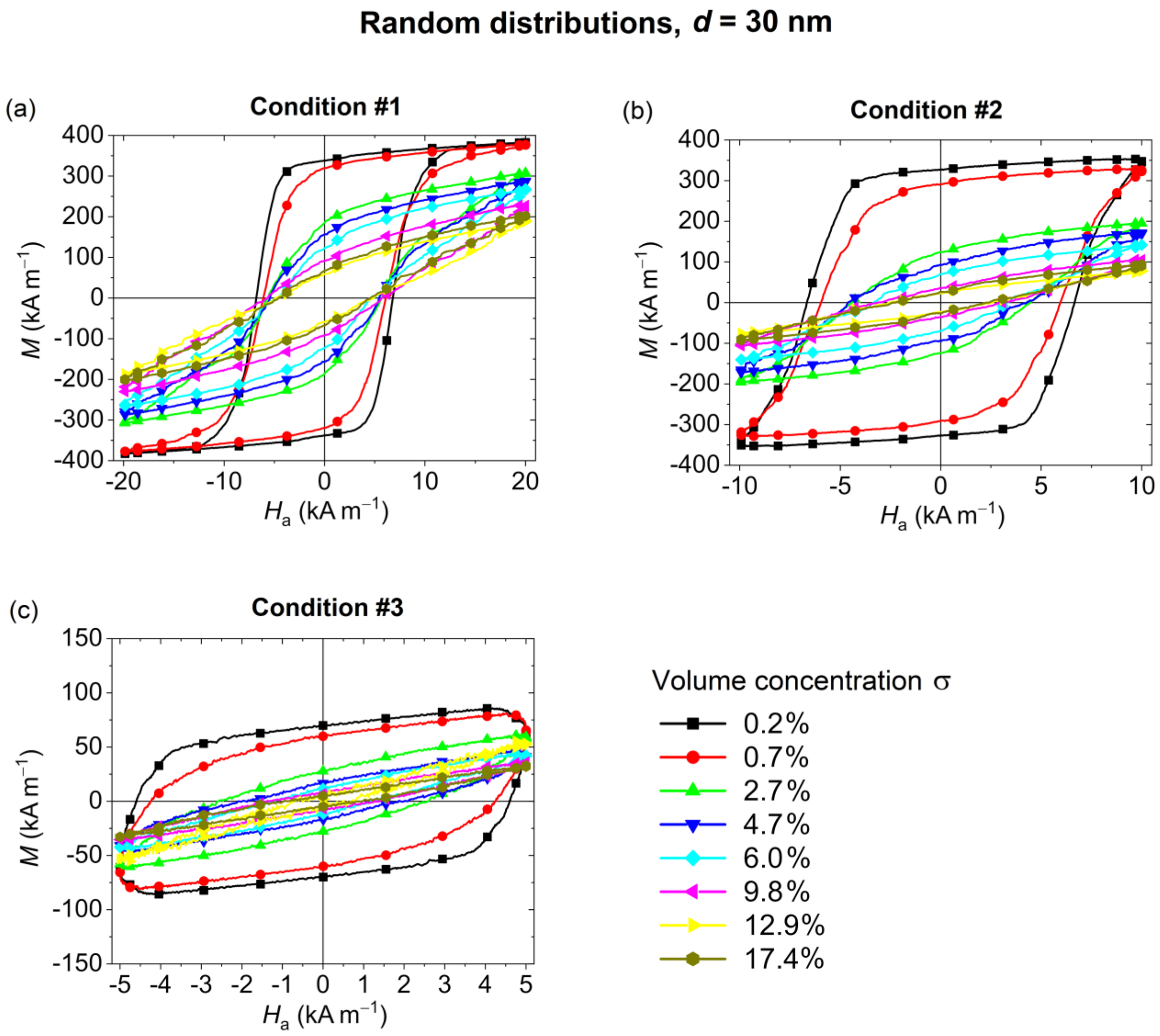

Fig. S9 Dynamic hysteresis loops versus volume concentration σ for interacting 30 nm sized Fe_3O_4 NPs randomly distributed in the space, calculated under three excitation conditions: #1 − $\hat{H}_a$ = 20 kA m^{-1}, f = 250 kHz; #2 − $\hat{H}_a$ = 10 kA m^{-1}, f = 500 kHz; #3 − $\hat{H}_a$ = 5 kA m^{-1}, f = 1 MHz.
